# Diagnostic comparison of Centers for Disease Control and Prevention and International Obesity Task Force criteria for obesity classification in South African children

**DOI:** 10.4102/phcfm.v9i1.1383

**Published:** 2017-08-31

**Authors:** Violet Kankane Moselakgomo, Marlise van Staden

**Affiliations:** 1 Department of Physiology and Environmental Health, School of Molecular and Life Science, University of Limpopo, South Africa

## Abstract

**Background:**

This study was designed to estimate overweight and obesity in school children by using contrasting definitions recommended by the Centers for Disease Control and Prevention (CDC) and the International Obesity Task Force (IOTF).

**Method:**

The sample size consisted of 1361 learners (*n* = 678 boys; *n* = 683 girls) aged 9–13 years who were randomly selected from Mpumalanga and Limpopo provinces of South Africa. A cross-sectional and descriptive design was used to measure the children’s anthropometric characteristics. Based on height and weight measurements, the children’s body mass index (BMI) was calculated and used to classify them as underweight, overweight and obese. Percentage body fat was calculated from the sum of two skinfolds (i.e. triceps and subscapular). Age-specific BMI, percentage body fat and sum of skinfolds were examined for the boys and girls.

**Results:**

A higher prevalence of overweight and obesity was found in boys and girls when the CDC BMI categories were used. In contrast, the IOTF BMI classifications indicated a strong prevalence of underweight among the children.

**Conclusion:**

In contrast to the IOTF index that yielded a greater occurrence of underweight among South African children, the CDC criteria indicated a higher prevalence of obesity and overweight among the same children. Future large-scale surveillance studies are needed to determine the appropriateness of different definitions in order to establish a more reliable indicator for estimating overweight and obesity in South African children.

## Introduction

Excessive body fatness has arguably become a paediatric health problem in developed nations. Excess body fat constitutes a serious risk to health^1^, affecting the physiological functions and physical fitness of an individual,^2,3,4,5,6^ and could negatively influence physical performance by having deleterious impact on mechanical, metabolic and thermoregulatory attributes of an activity.^7,8^ For example, in the United Kingdom, estimates of excessive childhood body fatness have increased from 2.0-fold to 2.8-fold in 10 years.^9^ Furthermore, in the United States, overweight or obese children are at risk of developing cardio-metabolic complications.^10,11^

The incidence of excessive body fatness is well documented among children and is now becoming a common feature in some developing nations such as India.^12^ There are concerns that a marked increase in the prevalence of body fatness, and consequently abdominal obesity, is one of the most common health problems with increasing incidence worldwide among people of all ages.^7,8^ For instance, results of the South African National Health and Nutrition Examination Survey 1^10^ indicate that overweight and obesity has been on the rise among children and adolescents, with a higher prevalence noted in girls than in boys (i.e. 16.5% and 7.1% vs 11.5% and 4.7%, for girls and boys, respectively).

Of particular concern is the level of central adiposity as demonstrated by high truncal body fat thicknesses, which is associated with the development of metabolic problems and cardiovascular diseases.^1^ In addition, there is substantial evidence that increase in the prevalence of abdominal obesity is probably intensified by the accompanying low physical fitness and inadequate physical activity levels.^6^ There is also a significant indication that the prevalence of abdominal obesity may manifest itself in adult life as a result of inadequate physical activity during childhood.

According to Mamabolo et al.^13^ the minimum percentage body fat considered safe and acceptable for children in good health is 5% in boys and 12% in girls. However, the percentage body fat varies considerably between boys and girls, physical fitness levels and age groups. Generally, boys have lower percentage body fat than girls. Subcutaneous fat is stored between the skin and the muscles, and between the muscles and the surrounding organs of the body.^14^ It represents about half of the fat in the human body, whereas the other half is located internally around other body organs.

Some studies have observed a correlation between central fat patterning and various disease risk factors including diabetes, high blood pressure and elevated lipid levels.^15,16,17,18^ Despite the positive correlation of excessive body fat with cardiovascular and metabolic health risk,^16,17^ there is still a lack of clarity as to which of the anthropometric measures best assesses body fatness, especially in children and adolescents.

In adults, a body mass index (BMI) of 30.0 kg/m^2^ has been set as the cut-off limit for the diagnosis of body fatness.^19^ However, producing a general cut-off value for children of all ages and sexes remains problematic, because the BMI varies with age regardless of gender. Nevertheless, studies have commonly used the BMI and waist-to-hip ratio as anthropometric indices to assess the pattern of fat distribution among children and adolescents.^20,21^ This classification system is recommended by the World Health Organization for all racial or ethnic groups. A number of classifications based on the BMI exist to determine people at risk of overweight and obesity. Typical among these are the Centers for Disease Control and Prevention (CDC) and International Obesity Task Force (IOTF) standards. Although use of the BMI is a convenient and low-cost method for assessing excessive or lean body fat, it may lack precision in terms of distinguishing between the contribution of muscles and fat mass. Firstly, this inaccuracy could result in the misclassification of children with regard to adiposity status. Secondly, the ability of these indices to reliably predict overweight and obesity among children has been questioned. Thirdly, in view of the possible uncertainty concerning the reliability of the classifications, the tendency exists that the health risks associated with excessive body fat and weight being investigated in children and adolescents may be misleading.

Skinfold thickness measures the fat located just underneath the skin (subcutaneous fat) and is a proxy indicator of total fat.^14^ Percentage body fat in children is estimated using the formula developed by Slaughter et al.^22^ based on measurements of triceps and subscapular skinfolds. Although excessive body fatness is considered an intermediary in the causal pathway from health to the development of cardiovascular disease,^7,8^ there are hardly any published data comparing the diagnostic accuracy of various anthropometric indices, such as the CDC BMI cut-off points and IOTF indices, to estimate body weight disorders among South African children. Therefore, the present study was designed to fill this gap by evaluating the appropriateness of two widely used definitions of obesity (i.e. the CDC BMI cut-off points and IOTF BMI classifications) to assess overweight and obesity among rural South African primary school children aged 9–13 years. The rural study locations were selected based on feasibility and logistical considerations. The study was guided by the hypothesis that the CDC and IOTF BMI classifications would yield discrepant estimates of obesity and overweight among the South African children. The adequacy of selection criteria for the assessment of obesity and overweight in children has practical applications in epidemiology and public health.

## Material and methods

### Study population and sample

From an estimated population of 12 800 learners, a total of 1361 children (*n* = 678 boys; *n* = 683 girls) aged 9–13 years were randomly selected from eight schools in each of Limpopo (*n* = 708) and Mpumalanga (*n* = 653) provinces. Class registers were used to draw a targeted sample of 100 children from each school. Based on the total number of learners per class, serial numbers were written on small pieces of paper, folded and placed in an empty paper box. The pieces of paper were scrambled and handpicked one after the other to select those who participated in the research.

### Anthropometric measurements

Stature, body mass and skinfolds (e.g. triceps and subscapular) were measured according to the protocol of the International Society for the Advancement of Kinanthropometry.^14^ Stature was measured to the nearest 0.1 cm in bare feet, with participants standing upright against a mounted stadiometer. A digital weighing scale (Tanita HD 309, Creative Products, MI, USA) calibrated regularly to the nearest 0.1 kg (after every 20 measurements) was used to measure body mass, with participants lightly dressed (e.g. underwear and T-shirt). Based on stature and body mass measurements, body composition indices including BMI (weight/height^2^) were derived using the following formula:

$$BMI=\frac{Mass(kg)}{{\left[Stature(m)\right]}^{2}}$$

BMI was used to classify the children in the following weight categories: underweight, overweight or obese for age and gender.

Overweight and obesity were measured by comparing BMI of the participants of this study with the CDC standard range of percentiles of BMI for age and gender. The CDC reference standard used the following cut-off points for classification: underweight (< 5th percentile), healthy weight (5th to < 85th percentile), overweight (85th to < 95th percentile) and obese (≥ 95th percentile). Specifically, the CDC growth norm defines children at risk of overweight and obesity if their BMI exceeds the 85th and 95th percentiles, respectively, for most routine assessments.

The IOTF cut-off points were used as a national standard for age and gender–specific norm of BMI classifications to categorise overweight and obesity in youths aged 2–18 years old. The IOTF benchmarks were determined through an extrapolation of the adult BMI cut-off points for obesity (30 kg/m^2^). Based on BMI data for nationally representative, cross-sectional surveys conducted in six countries (Brazil, Hong Kong, the Netherlands, Singapore, UK and the United States) that were fitted by the Lambda-Mu-Sigma (LMS: Lambda for the skew, Mu for the median, and Sigma for the generalised coefficient of variation) method, they linked BMI values at 18 years (16, 17, 18.5, 25 and 30 kg/m^2^ to children’s centiles, which were averaged across the countries.^15^

Triceps and subscapular skinfolds were measured to the nearest 0.1 mm using Rosscraft plastic slim guide calliper (Rosscraft, Canada). The subject was asked to stand erect with feet together, shoulders relaxed and arms hanging freely on the sides. The sums of two skinfolds [triceps (TSKF) and subscapular (SSKF)] were calculated and the Slaughter et al.^22^ equation was used to predict percentage body fat as follows:

(Triceps + Subscapular) > 35 mm):

Boys: % Body fat = 0.783 (TSKF + SSKF) + 1.6

Girls: % Body fat = 0.546 (TSKF + SSKF) + 9.7

(Triceps + Subscapular) < 35 mm):

Boys: % Body fat = 1.21 (TSKF + SSKF) – 0.008 (TSKF + SSKF)^2^ –1.7

Girls: % Body fat = 1.33 (TSKF + SSKF) – 0.065 (TSKF + SSKF)^2^ + 2.5

The anthropometric and body composition characteristics of the South African school children were compared with available normative data on South African children provided by the Human Sciences Research Council^10^ and CDC^8^ as well as those of other internationally published studies.

### Statistical analysis

The intra-observer reliability of anthropometric measurements was determined by examining technical error of measurement and intraclass correlation coefficient (Pearson’s method) based on data obtained from repeated measurements of a small sample of participants (*n* = 20). The intraclass correlation coefficients on anthropometric measures between observers were as follows: stature (0.98), body mass, (0.97), triceps (0.96) and subscapular (0.97). The reliability coefficients were within acceptable limits.

The participants’ anthropometric and body composition data were analysed using descriptive statistics (i.e. means and standard deviations). In order to compare the dependent variables across age and gender groupings, a series of two-way analyses of variance (ANOVA) (Age: 9–13 years; gender: boys and girls) were computed. Results of the ANOVA were used to test the major hypothesis designed for the study. For all statistical analysis, a probability level of 0.05 was taken to indicate significance.

### Ethical considerations

The Ethics Sub-Committee of the Faculty of Health Sciences, North-West University, South Africa (Ethics no: NWU-00088-12-S1), Provincial Heads of Education Departments, District Managers for the Department of Basic Education in Limpopo and Mpumalanga Provinces, and other relevant provincial regulatory bodies granted ethics approval for the research to be carried out. An information leaflet and informed consent forms were administered to the head teachers, pupils and their parents or guardians who also gave permission for the study to be conducted. In addition to parental consent, the children individually assented to participate in the research after the purpose and procedures were carefully explained to them in their native language. The children were particularly enthusiastic to participate in the research as they saw it as an interesting diversion from normal school work and an opportunity to play.

## Results

The study estimated body weight and fatness using CDC and IOTF classifications among 1361 primary school children (boys, *n* = 678; girls, *n* = 683) aged 9–13 years in Mpumalanga and Limpopo provinces of South Africa. Participants’ anthropometric measurements according to gender are presented in Table 1.

**TABLE 1:** Age-related distribution of anthropometric measurements of school children from Limpopo and Mpumalanga provinces of South Africa.

Age (years)	Limpopo Province					Mpumalanga Province				
	Sample size	Height (cm)	Weight (kg)	BMI	%Body fat	Sample size	Height (cm)	Weight (kg)	BMI	%Body fat
		M (s.d.)	M (s.d.)	M (s.d.)	M (s.d.)		M (s.d.)	M (s.d.)	M (s.d.)	M (s.d.)
9	126	130.45	26.72*	15.58*	28.44	82	130.90	28.63*	16.61*	33.84
		(7.06)	(5.24)	(1.87)	(1.71)		(6.68)	(4.75)	(1.67)	(2.93)
10	168	136.23*	31.77	16.31	21.47*	147	133.50*	30.87	15.88	26.25*
		(6.26)	(5.96)	(1.24)	(1.56)		(6.68)	(6.66)	(1.30)	(1.79)
11	215	139.51	34.48	17.58	16.41*	128	138.99	33.29	17.15	18.40*
		(6.58)	(10.70)	(4.69)	(1.74)		(6.84)	(6.05)	(2.41)	(1.67)
12	143	143.32	37.43	18.02	13.32	154	144.48	37.34	17.88	14.61
		(8.07)	(10.2)	(3.65)	(1.87)		(8.48)	(7.32)	(3.65)	(1.76)
13	56	147.11*	40.18*	18.36	11.94	142	151.43*	43.82*	19.06	12.47
		(7.75)	(9.47)	(2.95)	(1.70)		(8.48)	(8.39)	(3.22)	(1.94)

BMI, Body mass index; M, mean; s.d., standard deviation.

* *p* < 0.05.

The results showed that the school children from Mpumalanga Province had significantly higher mean values of percentage body fat at ages 10 (26.25 ± 1.79) and 11 (18.40 ± 1.67) years than their peers in Limpopo Province. However, non-significant differences were noted regarding BMI, except for the substantially higher value obtained for the 9-year-old Mpumalanga children (16.61 ± 1.67) in contrast to their counterparts from Limpopo Province (15.58 ± 1.87) (Table 1).

The results of the prevalence estimations for each BMI cut-off criteria provided by gender and age in Table 2 revealed that boys had higher prevalence of underweight when IOTF criteria (67.9%) were used, compared with CDC’s classification (4.68%).

**TABLE 2:** Prevalence of body mass index category for cut-off points criteria by gender and age.

BMI categories	9 yr *n* (%)	10 yr *n* (%)	11 yr *n* (%)	12 yr *n* (%)	13 yr *n* (%)	Total *n* (%)
**Boys** IOTF						
Underweight	100 (88.50)	130 (76.50)	120 (69.80)	83 (57.60)	31 (36.90)	464 (67.90)
Normal	13 (11.50)	38 (22.40)	48 (27.90)	52 (36.10)	45 (53.60)	196 (28.70)
Overweight	0 (0.00)	2 (1.20)	3 (1.70)	6 (4.20)	7 (8.30)	18 (2.60)
Obese	0 (0.00)	0 (0.00)	1 (0.60)	3 (2.10)	1 (1.20)	5 (0.70)
**CDC**						
Underweight	5 (4.42)	8 (4.70)	8 (4.65)	7 (4.86)	4 (4.80)	32 (4.68)
Normal	90 (79.60)	136 (80.00)	138 (80.20)	115 (79.70)	67 (79.80)	546 (79.86)
Overweight	12 (10.60)	17 (10.00)	17 (9.88)	14 (9.70)	8 (9.50)	68 (9.94)
Obesity	6 (5.31)	9 (5.29)	9 (5.23)	8 (5.50)	5 (5.95)	37 (5.46)
**Girls** IOTF						
Underweight	86 (90.50)	117 (80.70)	142 (83.00)	120 (78.40)	75 (65.80)	540 (79.60)
Normal	9 (9.50)	26 (17.90)	25 (14.60)	29 (19.00)	38 (33.30)	127 (18.70)
Overweight	0 (0.00)	1 (0.70)	2 (1.20)	3 (2.00)	1 (0.90)	7 (1.00)
Obese	0 (0.00)	1 (0.70)	2 (1.20)	1 (0.70)	0 (0.00)	4 (0.60)
**CDC**						
Underweight	4 (4.21)	7 (4.83)	8 (4.67)	7 (4.57)	5 (4.40)	31 (4.54)
Normal	75 (78.95)	116 (80.00)	137 (80.10)	122 (79.70)	91 (79.80)	541 (79.70)
Overweight	11 (11.60)	14 (9.65)	17 (9.97)	16 (10.50)	12 (10.50)	70 (10.40)
Obese	5 (5.30)	8 (5.52)	9 (5.26)	8 (5.23)	6 (5.26)	36 (5.31)

BMI, body mass index; CDC, Centers for Disease Control and Prevention; IOTF, International Obesity Task Force; n, number of participants; yr, years old.

Centers for Disease Control and Prevention’s classification indicated higher prevalence of overweight (9.94%) and obesity (5.46%) compared to the same categories using IOTF classification (2.6% overweight and 0.7% obesity). IOTF criteria also showed higher underweight prevalence (79.6%) in girls compared with the CDC’s definition (4.54%). Prevalence of overweight (10.4%) and obesity (5.31%) in girls was also higher when using CDC cut-off point in contrast to the IOTF criteria (1.0% overweight and 0.6% obesity) (Table 2).

## Discussion

The results of this study provide information on estimating body fatness using different definitions among rural primary school children aged 9–13 years from two South African provinces. Estimating body weight disorders using the CDC and IOTF BMI-based methods has become a hypothetical viewpoint. In previous studies, comparisons between countries were hindered by the use of diverse criteria for describing overweight and obesity.^23,24,25,26,27,28,29,30,31,32,33,34,35^ Furthermore, age- and gender-related BMI percentiles used for evaluating body weight disorders in children could be regarded as a right set of measure to screen an individual’s levels of obesity or thinness, thereby providing data that could be helpful to detect body weight related health problems. In a study carried out among a sample of Portuguese school age children, it was stated that the age- and gender-related BMI percentiles for screening for body weight disorders clearly distinguish BMI criteria for children.^6^

It is important to understand that the IOTF reference standard and the CDC norms do not produce the same outcome in terms of estimating the prevalence of underweight, overweight and obesity among children. This discrepancy could be because of the dissimilar methodologies used to determine cut-off criteria and sample selection. Goon et al.^8^ also emphasised that the adequacy of a sample could serve as a study’s strength when it comes to screening children for overweight and obesity because a huge sample size could produce noteworthy findings which may result in significantly different research outcomes.^8^ However, biased or non-representative samples could weaken and consequently confound the findings of studies.

The findings obtained in this study indicated that the IOTF benchmark yielded higher values of underweight, in contrast to the CDC classification that gave higher estimates in overweight and obesity for the combined samples of male and female South African children. The present findings are consistent with those of Goon et al.^8^ and Lobstein and Jackson-Leach^12^ who also reported a more conventional estimate of obesity and overweight compared with CDC norms. Furthermore, Kain et al.^11^ found lower body fat and weight estimates using IOTF criteria for the children aged 6–8 years old. The prevalence of overweight for boys was 23.3% using the CDC standards and 18.3% based on IOTF definition, while for obesity, these figures were 10.8% (CDC) and 7.8% (IOTF). Kain et al.^11^ stated further that the results found for boys were similar to those obtained for girls. In contrast, the CDC cut-off point demonstrated higher prevalence of overweight and obesity in girls compared to boys.

The findings of this study showed that there were significant gender differences in the IOTF and CDC estimates of underweight, overweight, and obesity in South African children. Previous research has also indicated similar gender-based discrepancies.^15^ In this study, the prevalence rates also revealed large dissimilarities across age categories. Particularly, the findings showed a growing increase in the incidence of overweight and obesity among boys and girls.^8,12^, However, the large differences between IOTF and CDC prevalence has been attributed to the procedures used for estimating overweight and obesity in previous research, which have included skinfold measurements, bioelectric impedance techniques and waist-to-hip ratio classifications.^4,11,25^

The fact that the low prevalence of weight disorders was observed using IOTF estimate calls for a reasonable level of caution not to neglect early diagnosis and epidemic prevention. Another important significance of CDC norm is the fact that it may assist in the early identification of a greater number of participants affected by body weight abnormalities, so as to reduce the risk of metabolic syndrome related to excessive weight increase.

The results of this study somewhat depict trends in the economic development of the Western world that is already at an advanced stage. By contrast, South Africa is a developing nation witnessing considerable socioeconomic changes and nutrition transition. It is not surprising therefore, that there are marked differences in the prevalence of overweight and obesity in the participants of the present study which are comparable with the relatively high estimates reported for children of similar age groups in developed countries.^10^ The Nigerian study by Goon et al.^8^ also reported a similar tendency.

However, it is interesting to note that while the South African children in the present study had higher percentage of overweight and obesity regardless of gender than Nigerian children, they were considerably less obese and overweight compared with American and Mexican children as indicated in the international comparison of statistics on overweight and obesity (Table 3).

**TABLE 3:** International comparison of statistics on overweight and obesity with the present study.*

Country	Prevalence of overweight (%)		Prevalence of obesity (%)		References
	Boys	Girls	Boys	Girls	
Australia	13.00	12.00	-	-	Wickramasinghe et al. (2005)^24^
Australia	27.00	27.00	11.00	12.00	Telfor et al. (2008)^25^
Australia	14.00	5.20	16.50	5.80	Wake et al. (2007)^26^
East Poland	15.00	15.80	3.60	3.70	Malecka-Tendera et al. (2005)^27^
Indian (Punjab)	12.24	14.31	5.92	6.27	Sidhu et al. (2006)^21^
Irish	14.90	15.90	9.20	14.30	O’Neill et al. (2007)^28^
Mexico	22.30	23.60	28.00	21.20	Del-Rio-Navarro et al. (2008)^29^
Spain	12.00	5.00	8.00	2.00	Montero (2005)^30^
Thailand	-	10.70	-	7.70	Pawloski et al. (2008)^31^
The Netherlands	23.40	5.20	30.20	7.20	Fredricks et al. (2005)^32^
United Arab Emirates	16.40	22.80	6.10	7.80	Al-Haddad et al. (2005)^33^
United Arab Emirates	19.20	13.10	19.80	12.40	Malik & Bakir (2007)^34^
USA	20.00	22.00	22.00	23.00	Dubose et al. (2006)^35^
Nigeria	2.10	3.20	1.60	2.80	Goon et al. (2010)^8^
South Africa	9.94	10.40	5.46	5.31	Present study*

*Source*: Goon et al. 2010

* CDC definition.

-, data not available.

The increased prevalence of overweight and obesity in South Africa children is not surprising. Like in the Western world, many South African children now have access to food rich in high calories such as snacks and those readily available in fast food outlets in many parts of the country.^10,13,16^ There is also a rise in technological advancement and automation that has reduced children’s involvement in physical activities.^18^ The present findings that showed a high level of underweight, more especially when IOTF criteria were used and to a lesser extent with the CDC benchmark, suggest that the children may be at the risk of malnutrition.

However, the major limitation of this study is that age-specific BMI values used in the data analysis may be misrepresentative of actual body sizes. This is probably because BMI is a product of the division of body weight by height squared, which might not precisely show the risk of underweight individuals who are short and muscular. Many underfed children seem to come from a low resourced upbringing and may not have regular meals. Therefore, the growing prevalence of childhood underweight, noted in the present study and obesity reported in other studies, may be anticipated to influence the prevalence levels at adulthood.

As the choice of which cut-off points to use in the definition of childhood overweight and obesity is debatable it is expedient to devise measures to identify the growing trend of overweight and obesity among children. Specifically, it is important for experts in epidemiology and public health to develop national, population-specific reference standards and cut-off points in order to reliably quantify overweight and obesity. In addition, policymakers may need to re-examine current methods that are being used to reverse the growing trends of body weight abnormalities among children and adolescents.

## Conclusions

In conclusion, this study indicated a considerably higher level of underweight in South African school children when IOTF criteria were used. Specifically, 67.9% of boys and 79.6% of girls were classified as underweight. In contrast to IOTF benchmark, the CDC standards yielded higher prevalence of overweight (boys: 9.94%; girls: 10.4%) and obesity (boys: 5.46%; girls: 5.31%) among the children. Therefore, these results support the hypothesis that the CDC and IOTF BMI classifications would yield discrepant estimates of obesity and overweight among the South African children. In view of the divergent estimates of the children’s body weight and fat characteristics found in this study, it is important that large-scale surveillance studies carried out to estimate body weight disorders among children should provide clear rationale for the choice of cut-off points. This is necessary to minimise errors that could confound data analysis and interpretation, consequently questioning the reliability and validity of such estimates.

## References

[1] American College of Sports Medicine Health-related physical fitness assessment manual. Philadelphia, PA: Lippincott Williams and Wilkins; 2005.

[2] American Heart Association Cardiovascular disease statistics [homepage on Internet]. 2010 [cited 2016 Nov 4]. Available from: http://www.circ.ahajournals.org

[3] Bailey R Evaluating the relationship between physical education, sport and social inclusion. Educ Rev. 2005;57(1):71–90. https://doi.org/10.1080/0013191042000274196

[4] Centre for Disease Control and Prevention National Health and Nutrition Education Survey/National Youth Fitness Survey (NHANES/NNYFS). Atlanta, GA: Centres for Disease Control and Prevention; 2010.

[5] Cole TJ, Flegal KM, Nicholls D, Jackson AA Body mass index cut offs to define thinness in children and adolescents: International survey. BMJ. 2007;335(7612):194 https://doi.org/10.1136/bmj.39238.399444.55 17591624 10.1136/bmj.39238.399444.55 PMC1934447

[6] De Sousa Lopes HM Diagnostic accuracy of CDC, IOTF and WHO criteria for obesity classification, in a Portuguese school-aged children population. Porto, Portugal: Porto Institute of Public Health, University of Porto; 2012.

[7] Draper CE, Basset S, De Villiers A, Lambert EV Results from South Africa’s 2014 report card on physical activity for children and youth. J Phys Act Health. 2014;11(Suppl 1):S98–104. https://doi.org/10.1123/jpah.2014–0185 25426923 10.1123/jpah.2014-0185

[8] Goon DT, Toriola AL, Shaw BS Screening for body weight disorders in Nigerian children using contrasting definitions. Obes Rev. 2010;11(7):508–515. https://doi.org/10.1111/j.1467-789X.2009.00682.x 19874528 10.1111/j.1467-789X.2009.00682.x

[9] Hajian-Tilaki K, Heidari B A comparison between international obesity task force and centre for disease control references in assessment of overweight and obesity among adolescents in Babol, Northern Iran. Int J Prev Med. 2013; 4(2):226. 23543826 PMC3604857

[10] Hoosain E, Dwane N, Reddy P South African national health and nutrition examination survey. Pretoria: Human Sciences Research Council; 2013.

[11] Kain J, Uauy R, Vio F, Albala C Original communications-trends in overweight and obesity prevalence in Chilean children: Comparison of three definitions. Eur J Clin Nutr. 2002;56(3):200–204. https://doi.org/10.1038/sj.ejcn.1601301 11960294 10.1038/sj.ejcn.1601301

[12] Lobstein T, Jackson-Leach R Child overweight and obesity in the USA: Prevalence rates according to IOTF definitions. Int J Pedtr Obes. 2007;2(1):62–64. https://doi.org/10.1080/17477160601103948 10.1080/17477160601103948 17763012

[13] Mamabolo RL, Kruger HS, Lennox A, et al Habitual physical activity and body composition of black township adolescents residing in the North West Province: South Africa. Pub Health Nutr. 2007;10(10):1047–1056. 17381956 10.1017/S1368980007668724

[14] Marfell-Jones MJ, Stewart AD, De Ridder JH International standards for anthropometric assessment. Potchefstroom, South Africa: International Society for the Advancement of Kinanthropometry 2006.

[15] Monasta L, Lobstein T, Cole TJ, Vignerová J, Cattaneo A Defining overweight and obesity in preschol children: IOTF reference or WHO standard? Obes Rev. 2011;12(4):295–300. https://doi.org/10.1111/j.1467-789X.2010.00748.x 20492539 10.1111/j.1467-789X.2010.00748.x

[16] Monyeki KD, Kemper HC, Makgae PJ The association of fat patterning with blood pressure in rural South African children: The Ellisras Longitudinal Growth and Health Study. Int J Epidemiol. 2006;35(1):114–120. https://doi.org/10.1093/ije/dyi219 16260449 10.1093/ije/dyi219

[17] Monyeki MA, Neetens R, Moss SJ, Twisk J The relationship between body composition and physical fitness in 14 year old adolescents residing within the Tlokwe local municipality, South Africa: The PAHL study. BMC Pub Health. 2012;12(1):1 https://doi.org/10.1186/1471-2458-12-374 22626033 10.1186/1471-2458-12-374 PMC3461487

[18] Monyeki MA Physical activity and health in children: How much do we know? Afr J Phys Health Educ Rec Dance. 2014;20(2:1):323–342.

[19] Ogden CL, Carroll MD, Curtin LR, Lamb MM, Flegal KM Prevalence of high body mass index in US children and adolescents, 2007–2008. JAMA. 2010;303(3):242–249. https://doi.org/10.1001/jama.2009.2012 20071470 10.1001/jama.2009.2012

[20] Quinns E Body composition versus body fat. Sports Medicine Newsletter, 2006 April 10, pp. 4–30.

[21] Sidhu S, Kaur N, Kaur R Overweight and obesity in affluent school children of Punjab. Ann Hum Biol. 2006;33(2):255–259. https://doi.org/10.1080/03014460600578631 16684697 10.1080/03014460600578631

[22] Slaughter MH, Lohman TG, Boileau R, et al Skinfold equations for estimation of body fatness in children and youth. Hum Biol. 1988;60:709–723. 3224965

[23] Toriola AL, Moselakgomo VK, Shaw BS, Goon DT Overweight, obesity and underweight in rural black South African children. S Afr J Clin Nutr. 2012;25(2): 57–61.

[24] Wickramasinghe VP, Cleghorn GJ, Edmiston KA, Murphy AJ, Abbott RA, Davies PSW Validity of BMI as a measure of obesity in Australian white Caucasian and Australian Sri Lankan children. Ann Hum Biol. 2005;32:60–71. 15788355 10.1080/03014460400027805

[25] Telford RD, Cunningham RB, Daly RM et al Discordance of international adiposity classifications in Australian boys and girls - the LOOK study. Ann Hum Biol. 2008; 35:334–341. 18568596 10.1080/03014460802014625

[26] Wake M, Hardy P, Canterford L, Sawyer M, Carlin JB Overweight, obesity and girth of Australian preschoolers: prevalence and socio-economic correlates. Int J Obes. 2007;31:1044–1051. 10.1038/sj.ijo.0803503 17146451

[27] Malecka-Tendera E, Klimelk K, Matusik P, OlszaneckaGlinianowicz M, Lehingue Y Obesity and overweight prevalence in Polish 7- to 9-year-old children. Obes Res. 2005;13:964–968. 15976137 10.1038/oby.2005.112

[28] O’Neill JL, McCarthy SN, Burke SJ et al Prevalence of overweight and obesity in Irish school children, using four different definitions. Eur J Clin Nutr. 2007;61: 743–751. 17180155 10.1038/sj.ejcn.1602580

[29] Del-Rio-Navarro BE, Velazquez-Monroy O, Lara-Esqueda A et al Obesity and metabolic risks in children. Arch Med Res. 2008;39:215–221. 18164967 10.1016/j.arcmed.2007.07.008

[30] Montero P Nutritional and diet quality of visually impaired Spanish children. Ann Hum Biol. 2005;32:1294–1296. 10.1080/03014460500142744 16147398

[31] Pawloski LR, Ruchiwit M, Pakapong Y A cross-sectional examination of growth indicators from Thai adolescent girls: evidence of obesity among Thai youth? Ann Hum Biol. 2008;35:378–385. 18608112 10.1080/03014460802082119

[32] Fredriks AM, Buuren SV, Hira Sing RA, Wit JM, VerlooveVanhorick SP Alarming prevalences of overweight and obesity for children of Turkish, Moroccan and Dutch origin in the Netherlands according to international standards. Acta Paediatr. 2005;94:496–498. 16092466 10.1111/j.1651-2227.2005.tb01923.x

[33] Al-Haddad F, Little BB, Abdul Ghafoor AGM Childhood obesity in United Arab Emirates school children: a national study. Ann Hum Biol. 2005;32:72–79. 15788356 10.1080/03014460400027425

[34] Malik M, Bakir A Prevalence of overweight and obesity among children in the United Arab Emirates. Obes Rev. 2007;8:15–20. 17212792 10.1111/j.1467-789X.2006.00290.x

[35] Dubose KD, Stewart EE, Charbonneau SR, Mayo MS, Donnelly JE Prevalence of the metabolic syndrome in elementary school children. Acta Paediatr. 2006;95:1005–1011. 16882578 10.1080/08035250600570553

